# Alteration of Gut Microbiome and Correlated Lipid Metabolism in Post-Stroke Depression

**DOI:** 10.3389/fcimb.2021.663967

**Published:** 2021-04-22

**Authors:** Wenxia Jiang, Lei Gong, Fang Liu, Yikun Ren, Jun Mu

**Affiliations:** Department of Neurology, The First Affiliated Hospital of Chongqing Medical University, Chongqing, China

**Keywords:** post-stroke depression, gut microbiome, metabolome, metabolic pathways, lipid metabolism

## Abstract

**Background:**

The pathogenesis of post-stroke depression (PSD) remains largely unknown. There is growing evidence indicating that gut microbiota participates in the development of brain diseases through the gut-brain axis. Here, we aim to determine whether and how microbial composition and function altered among control, stroke and PSD rats.

**Materials and Methods:**

After the PSD rat model was successfully established, gut microbiome combined with fecal metabolome approach were performed to identify potentially PSD-related gut microbes and their functional metabolites. Then, correlations between behavior indices and altered gut microbes, as well as correlations between altered gut microbial operational taxonomic units (OTUs) with differential metabolites in PSD rats were explored. Enrichment analysis was also conducted to uncover the crucial metabolic pathways related to PSD.

**Results:**

Although there were some alterations in the microbiome and metabolism of the control and stroke rats, we found that the microbial and metabolic phenotypes of PSD rats were significantly different. The microbial composition of PSD showed a decreased species richness indices, characterized by 22 depleted OTUs mainly belonging to phylum *Firmicutes*, genus *Blautia* and *Streptococcus*. In addition, PSD was associated with disturbances of fecal metabolomics, among them Glutamate, Maleic acid, 5-Methyluridine, Gallocatechin, 1,5-Anhydroglucitol, L-Kynurenine, Daidzein, Cyanoalanine, Acetyl Alanine and 5-Methoxytryptamine were significantly related to disturbed gut microbiome (P ≤ 0.01). Disordered fecal metabolomics in PSD rats mainly assigned to lipid, amino acid, carbohydrate and nucleotide metabolism. The steroid biosynthesis was particularly enriched in PSD.

**Conclusions:**

Our findings suggest that gut microbiome may participate in the development of PSD, the mechanism may be related to the regulation of lipid metabolism.

## Introduction

Post-stroke depression (PSD) is a common mood disorder, which often indicates a poor prognosis (Kutlubaev and Hackett, 2014) and high mortality (Bartoli et al., 2013). At any point within five years of the stroke, approximately one-third of stroke survivors have PSD (Hackett and Pickles, 2014). The hypotheses regarding the pathogenesis of PSD, include psychosocial distress, alteration of monoamine neurotransmitter, activation of the hypothalamic-pituitary-adrenal axis and disturbed energy metabolism (Villa et al., 2018). However, the specific pathophysiology of PSD remains unknown.

Gut microbiome is the major microbial community that settles in the human body, and affects the host’s nutrition, metabolism and immune function (Ghaisas et al., 2016). Previous studies found that altered gut microbiome has been implicated in stroke and depression (Zheng et al., 2016; Zhu et al., 2016; Chen et al., 2019; Lee et al., 2020; Zheng et al., 2020). The microbial metabolites are also associated with depression and stroke. A previous study showed that depressed mice were characterized by disturbances in carbohydrate and amino acid metabolism (Zheng et al., 2016). Another study demonstrated that stroke was related to disturbances in amino acid and lipid metabolism (Yamashiro et al., 2017; Chen et al., 2019). Accumulating data indicated that the gut microbiota communicates with the central nervous system, influencing brain function and behavior through the microbiota-gut-brain axis (Cryan and Dinan, 2012; Cryan et al., 2019). Zhu et al. revealed that gut microbes can promote thrombosis by producing trimethylamine N-oxide (TMAO), thereby increasing the incidence of stroke (Zhu et al., 2016). Another study found a reduced fecal level of short chain fatty acids (SCFAs) in aged stroke mice, as a result, transplantation of fecal microbiota rich in SCFAs promoted the stroke recovery (Lee et al., 2020). However, so far, the gut microbiome and microbial metabolism of PSD rats have not been explored.

In order to determine whether the microbial composition and function of PSD rats have changed, and how they may have changed, 16S ribosomal RNA (rRNA) gene sequencing technology was firstly applied to identify alteration of gut microbiome compositions. Then, the changes of metabolites in the fecal samples of PSD rats were captured by gas chromatography-mass spectrometry (GC-MS), before the correlation between gut microbes and bacterial metabolites was revealed.

## Materials and Methods

### Animals

**Figure 1:**
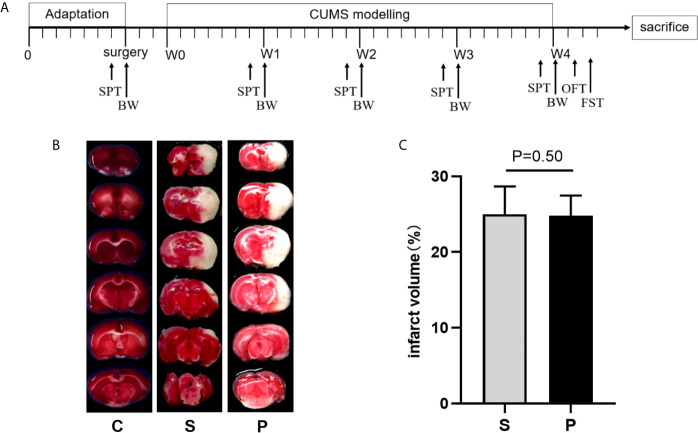
Time schedule of experimental procedures and TTC-stained brain sections. **(A)** Time schedule of experimental procedures. CUMS, chronic unpredictable mild stress; W0, Beginning of CUMS; W1–W4, CUMS was performed as described in the materials and methods section for 4 weeks; BW, body weight; SPT, sucrose preference test; OFT, open field test; FST, forced swimming test. **(B)** The representative TTC-stained brain sections. **(C)** Quantification of infarction volumes was calculated based on TTC staining (n = 6 per group, P > 0.05). C, Control; S, Stroke; P, post-stroke depression.

Male Sprague–Dawley (SD) rats about 180-200g were purchased from the Experimental Animal Center of Chongqing Medical University (Chongqing, China). All animal procedures were approved by the Ethics Committee of Chongqing Medical University (Permit No. 2020–459) and complied with the guidelines of Animal Use and Care of the National Institutes of Health. The workflow is displayed in **Figure 1A**. The rats were housed in groups of five per cage, ad libitum food and water. The room was maintained in 12-hour light/dark cycle (lights on at 8:00 a.m.), with constant temperature of 21–22°C and humidity of 50 ± 5%. They were allowed to acclimatize for 1 week before sucrose preference test (SPT) was performed at baseline. At the same time, they were trained to consume 1% sucrose solution for taste adaptation. 86 rats with similar baseline performance were included. There were 14 rats in the control group, and 72 rats receiving ischemia-reperfusion (IR) surgery. Animals that underwent IR surgery and survived 24 hours later were randomly divided into the stroke group (n = 21) and the PSD group (n = 30). Three days after surgery, rats in the PSD group were housed in a single cage and subjected to a chronic unpredictable mild stress (CUMS) regimen for 4 weeks.15 rats died within 24hrs after surgery. Six rats without neurological symptoms were excluded. In view of the protective effect of estrogen on ischemic injury, only male rats were used in this study (Xu et al., 2018).

### Focal Cerebral Ischemia and Reperfusion Surgery

As described by Belayev, et al, the FCIR model was established by transient middle cerebral artery occlusion (MCAO) (Belayev et al., 1996). Briefly, rats were anesthetized *via* intraperitoneal injection of 3.5% chloral hydrate (350 mg/kg). A midline incision was made on the neck, the right common carotid artery (CCA), external carotid artery (ECA) and internal carotid artery (ICA) were exposed. A nylon monofilament suture (diameter 0.26 mm, Beijing Xinong Technology Co. Ltd., China) with a slightly enlarged round tip (diameter 0.34–0.36 mm) was gently advanced from the ECA into the lumen of the ICA, until it reached and occluded the middle cerebral artery (MCA). The distance from bifurcation of CCA to the tip of the suture inserted to occlude MCA, averaged 18–20 mm. After 2 hrs of ischemia, the suture was carefully removed to establish reperfusion. The body temperature was maintained at 36.5-37.5°C by a heating pat after the surgery. Animals in the control group underwent the same surgical procedure without suture insertion.

### Measurement of Cerebral Infarct Volume

Rats were euthanized on the 3rd day after reperfusion. The brains were removed and frozen for 30 min at −20°C. The forebrain was cut into consecutive 2 mm-thick coronal slices, stained with 2% 2,3,5-triphenyltetrazolium chloride (TTC, Sigma-Aldrich, USA) at 37°C for 10 min. The slices were photographed and image analysis software (ImagePro Plus 6.0, Media Cybernetics Co. USA) was used. The percentage of infarct volume was calculated *via* the following formula: right hemisphere infarct volume/total volume ×100% (Ikeda-Matsuo et al., 2006).

### Chronic Unpredictable Mild Stress

The CUMS regimen used in this study was adapted from Wang, et al. (2009), with minor modifications. Details were shown in **Supplementary Table S1**. Briefly, 1-2 of the following stressors were arranged in a random order daily: water deprivation for 24 hrs and followed by a sucrose preference test, food deprivation for 24 hrs, swimming in 4°C water for 5 min, tail pinch for 1 min, 45° cage tilt for 24 hrs, soiled cage (200mL water in 100g padding) for 24 hrs, overnight illumination (lights on for a total of 36 hrs), restrained with steel wire tube for 4 hrs (well-ventilated, rats were not able to move forward or backward in tubes).

### Behavioral Tests

All behavioral tests were performed by well-trained and experienced observers who were blind to the grouping of the animals.

#### Neurological Deficit Scoring Evaluation

The neurological deficits of the animals were evaluated at 24hrs after the IR surgery with the 5-point neurological scale according to Longa et al. (1989). Specifically, score 0, no neurologic deficit; score 1, failure to extend left forepaw fully when held by tails; score 2, circling to the left side; score 3, falling to the left side; score 4, no walk spontaneously with depressed level of consciousness. Rats with a score of 0 or 4 were removed from further study.

#### Sucrose Preference Test

SPT was used to assess anhedonia of rats (Guo et al., 2009). Before testing, animals were water deprived for 24 hrs. One bottle of purified water and another bottle of 1% sucrose solution were provided to each rat for 1 hr. The positions of two bottles will be switched after 30 min. SPT was performed before surgery and once a week during CUMS. Sucrose preference rate was calculated by sucrose intake (g)/[sucrose intake (g) + water intake (g)].

#### Open Field Test

OFT was used to evaluate the locomotor activity, overall exploratory and anxiety of rats in a new environment (Choleris et al., 2001; Niu et al., 2015). OFT was performed at the end of CUMS. The experimental device consists of a box (100 cm × 100 cm × 40 cm) and an automatic data acquisition and processing system (SMART 3.0, Panlab, Spain). Each rat was placed at the center of the box and monitored for 5 min. The total distance of spontaneous moves and percentage of duration time spent in the center square (duration = time spent in the center square (s)/total time (s) × 100%) were videotaped and quantified by a video-computerized tracking system. Each rat was tested individually and only once. The box was cleaned thoroughly before each animal was tested.

#### Forced Swimming Test

FST was used to assess behavioral despair of rodent (Cryan et al., 2005). FST was performed at the end of CUMS. Each rat was placed in a plastic cylinder (60 cm high, 20 cm diameter) with water (21–23°C, 30 cm in depth) for 6 min. The last 4 min of immobility time was recorded by a video-computerized tracking system (SMART 3.0, Panlab, Spain).

#### Body Weight Measurement and Fecal Samples Collection

Body weight measurement and fecal samples collection were performed before surgery and once a week during CUMS. Fecal samples were collected by lifting tail and immediately frozen in liquid nitrogen and stored at −80°C.

### Deoxyribonucleic Acid Extraction, Polymerase Chain Reaction Amplification, and Illumina MiSeq Sequencing

Total bacterial DNA was extracted from fecal samples using the OMEGA-soil DNA Kit (Omega Bio-Tek, USA) according to the manufacturer’s protocols. The V3-V4 region of the bacteria 16S rRNA gene was targeted and PCR amplified with primers 338F (5’-ACTCCTACGGGAGGCAGCAG-3’) and 806R (5’-GGACTACHVGGGTWTCTAAT-3’). The PCR cycling conditions were as follows: 95°C for 3 min, followed by 27 cycles of 95°C for 30 s, 55°C for 30 s, 72°C for 45 s, and a final extension at 72°C for 10 min. PCR reactions were performed in triplicate 20μL mixture. Amplicons were extracted from a 2% agarose gel and further purified with the AxyPrep DNA Gel Extraction Kit (Axygen Biosciences, Union City, CA, USA) and quantified using QuantiFluo-ST (Promega, USA). Purified amplicons were paired-end sequenced (2 × 300) on an Illumina MiSeq platform (Illumina, San Diego, USA) according to the standard protocols.

### 16S rRNA Gene Sequence Analysis

Raw fastq files were demultiplexed, and quality-filtered using QIIME (version 1.17, http://qiime.org/). Truncate the 250bp reads at any site of more than three sequential, the average quality score < 20 were accepted. Reads shorter than 50 bp containing barcode/primer errors or ambiguous base calls were discarded. Chimeric sequences were identified and removed using UCHIME (http://drive5.com/uchime). Operational taxonomic units (OTUs) were clustered with 97% similarity cutoff using UPARSE (version 7.1 http://drive5.com/uparse/). Species diversity indices (Shannon, Simpson) and species richness indices (Ace and Chao) were used to evaluate α-diversity. Principal co-ordinate analysis (PCoA) was used to visually evaluate the whole difference and similarity of bacterial communities among control, stroke and PSD group (n=6 per group) (Zhang et al., 2019). The key bacterial taxa responsible for discrimination among the three groups were identified with linear discriminant analysis effect size (LEfSe) analysis (Segata et al., 2011). LEfSe analysis was used to identify the different OTUs by calculating the effect of the abundance of each OTU (LDA > 2.0 and P value ≤ 0.05).

### Fecal Metabolome Analysis

Fecal metabolome analysis was performed as previously described (Chi et al., 2017; Zheng et al., 2019). Briefly, fecal samples were processed under anaerobic conditions. Each fecal sample (100 mg) was transferred into 1.5 mL eppendorf tubes with reduced sterile phosphate buffered saline and an equal volume of donor suspension was used to prepare pool. Homogenized samples were extracted put in -20°C for 30 min, and then were centrifuged at 13000 rpm, 4°C for 15 min. Supernatant was dried with a centrifugal freeze dryer, then the dried extracts were used to derivatization. Gas chromatography-mass spectrometry (GC/MS; Agilent 7890A/5975C, CA, USA) was used to characterize the fecal metabolome analysis. The GC/MS three-dimensional matrices comprised of sample names (observations), peak indexes (RT-m/z pairs), and normalized peak area percentages were imported into a SIMCA (version14.0, Umetrics, Umeå, Sweden). Orthogonal Partial Least Squares Discrimination Analysis (OPLS-DA) was used to visually discriminate the PSD subjects from control and stroke (n=8 per group). By analysis of Principal Component Analysis (PCA) loadings, the differential metabolites responsible for discriminating among the three groups were identified (variable importance plot (VIP) > 1.0, and p-values < 0.05). Pathway analyses were carried out based on Kyoto Encyclopedia of Genes and Genomes (KEGG) Pathway Database and conducted by MetaboAnalyst 4.0.

### Statistical Analysis

Statistical analyses were carried out using SPSS version 19 (SPSS, Chicago, IL, US). Continuous variables such as sucrose preference, total distance and center time of OFT, immobility time in FST and body weight were analyzed by one-way analysis of variance (ANOVA). The results were presented as mean ± standard error of the mean (SEM) unless otherwise indicated. The least significant difference (LSD) post-hoc test was used to find out which two groups differed significantly if a significant difference was observed in ANOVA. The α-diversity was analyzed by Kruskal-Wallis Test. Beta-diversity was analyzed by Adonis analysis. The cerebral infarct volume between stroke and PSD was analyzed by student t-test, statistical result was presented as mean ± SEM. The non-parametric test was used when appropriate. Spearman correlation analysis was used to determine the correlation between the behavior indices of PSD and the discriminative OTUs, as well as the correlation between changes in gut microbes and the main differential fecal metabolites in PSD. The multiple testing corrections were conducted using Benjamini and Hochberg False Discovery Rate. Statistical significance level was set at p < 0.05.

## Results

### Behavioral Characteristics of the PSD Rats

**Figure 2:**
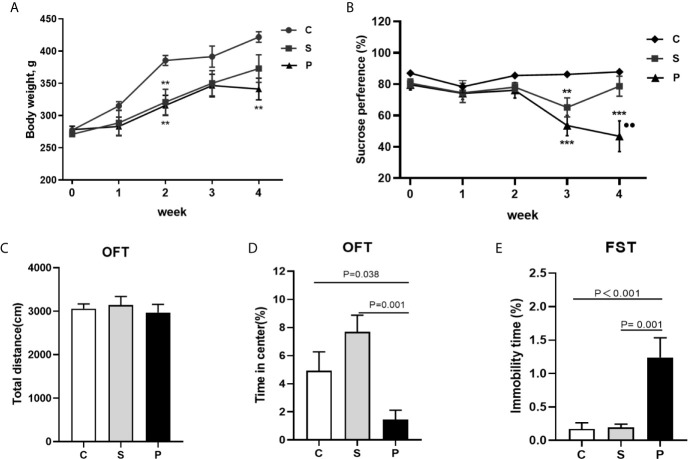
Body weight and behavioral tests. **(A)** Body weight during CUMS of three group (n = 8 per group); **(B)** Sucrose preference during CUMS of three group (n = 8 per group); **(C)** The total distance of OFT was no significant difference among 3 groups after a 4-week CUMS exposure (n = 8 per group); **(D)** Percentage of duration time spent in the center square of OFT was compared among 3 groups after a 4-week CUMS exposure (n = 8 per group); **(E)** Immobility time comparison of FST among 3 groups after a 4-week CUMS exposure (n = 8 per group); C, Control; S, Stroke; P, PSD; PSD group vs. control group, **P < 0.01 and ***P < 0.001; PSD group vs. stroke group, ●● P < 0.01; SPT, sucrose preference test; OFT, Open field test; FST, Forced swimming test.

Before the initiation of CUMS, the cerebral infarct volume showed no significant difference between the groups (**Figures 1B, C**; n = 6 per group, P > 0.05). Compared with control group, PSD rats were characterized by decreased sucrose preference and body weight on 4th week of CUMS (**Figures 2A, B**; n = 8 per group, P<0.01). There was no significant difference in the total distance of OFT among the three groups (**Figure 2C**; n = 8 per group, P > 0.05). But, compared to control and stroke group, PSD rats showed significantly decreased center time (**Figure 2D**; n = 8 per group). Meanwhile, compared to control and stroke rats, PSD rats showed significantly decreased immobility time in FST (**Figure 2E**; n = 8 per group). These results suggested that our PSD modeling was successful and depressive phenotype was independent of infarct volume.

### Decreased Species Richness Indices (Ace and Chao) in PSD

**Figure 3:**
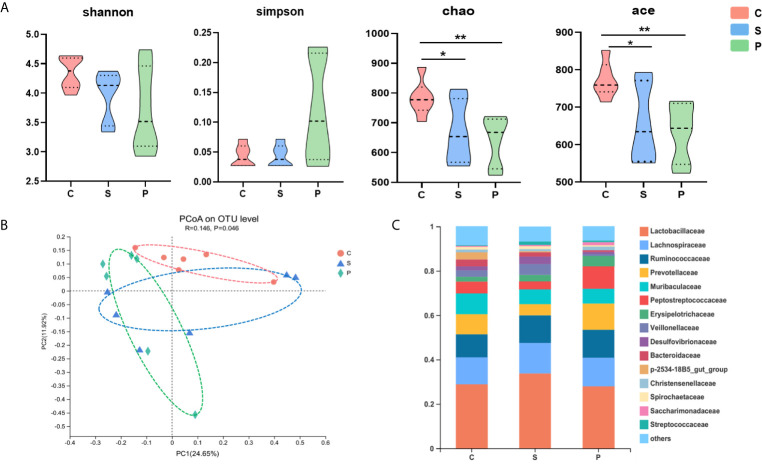
Gut microbial characteristics of control, stroke and PSD. **(A)** α-phylogenetic diversity analysis showing that PSD subjects were characterized by lower microbial richness in two indexes (Ace, Chao) relative to controls (n=6 per group), *P < 0.05, **P < 0.01. **(B)** At the OTU level, principal co-ordinates analysis (PCoA) showed gut microbial composition of rats with PSD was significantly different from that in control and stroke (n = 6 per group). **(C)** Relative abundance of gut microbes at the family level.

The 16S rRNA gene sequencing method was used to compare the fecal microbial composition of PSD, control and stroke. In the discovery set, we obtained 2117702 high-quality reads across all samples, with an average length of 418.13. These reads were clustered into 1410 OTUs at 97% sequence similarity. The α-diversity values including species diversity indices (Shannon and Simpson) and species richness indices (Ace and Chao) were compared among the PSD, control and stroke groups. We found that microbial richness indices were significantly different among three groups. Compared to control, the PSD rats were characterized by decreased Chao, Ace indices (**Figure 3A**, p < 0.01), suggesting decreased species richness indices in PSD.

### Alternations of Microbial Composition in PSD Rats

**Figure 4:**
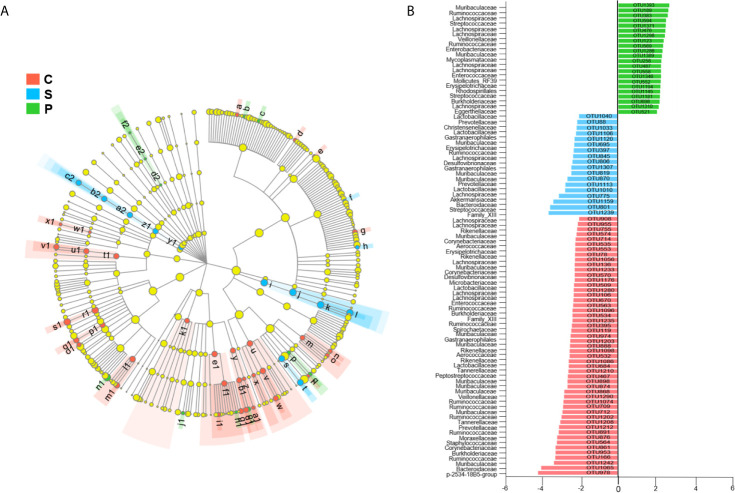
Linear discriminant analysis effect size (LEfSe) analysis**** (LDA > 2.0). Cladogram **(A)** and histogram **(B)** illustrated 91 OTUs responsible for discriminating the PSD, stroke and control groups. Compared to stroke and control groups, the PSD rats were characterized by 22 discriminative OTUs (n = 6 per group).

To determine whether PSD was associated with altered microbial composition, β-diversity analysis was performed. PCoA revealed that the gut microbial composition of rats with PSD was significantly different from that in control and stroke (**Figure 3B**; n = 6 per group). The relative abundance of gut microbes at the OTU level was shown in **Figure 3C**. In order to identify the microbial characteristics that can distinguish PSD from control and stroke, the LEfSe analysis method was used to analyze the differential OTU among the three groups (**Figures 4A, B**). In total, 91 differentially abundant OTUs were identified. The stroke rats were characterized by 18 OTUs, mainly belonging to *Muribaculaceae* (3 OTUs), *Prevotellaceae* (2 OTUs), *Lachnospiraceae* (2 OTUs), *Lactobacillaceae* (2 OTUs), *Christensenellaceae* (1 OTUs), *Erysipelotrichaceae* (1 OTUs), *Ruminococcaceae* (1 OTUs), *Desulfovibrionaceae* (1 OTUs), *Akkermansiaceae* (1 OTUs), *Bacteroidaceae* (1 OTUs) and *Streptococcaceae* (1 OTUs) at the family level (**Figure 4B**). Compared with control and stroke, the PSD were characterized by 22 OTUs, mainly belonging to *Firmicutes* (14 OTUs), *Proteobacteria* (3 OTUs), *Bacteroidetes* (2 OTUs), *Tenericutes* (2 OTUs), and *Actinobacteria* (1 OTU) at the phylum level. 22 OTUs particularly overrepresented in PSD were assigned to the families of *Lachnospiraceae* (6 OTUs), *Lactobacillaceae* (1 OTU), *Streptococcaceae* (2 OTUs), *Erysipelotrichaceae* (1OTU), *Ruminococcaceae* (2 OTUs), *Veillonellaceae* (1 OTU), *Enterococcaceae* (1 OTU), *Muribaculaceae* (2 OTUs), *Burkholderiaceae* (1 OTU), *Enterobacteriaceae* (1 OTU), *Mycoplasmataceae* (1 OTU) and *Eggerthellaceae* (1 OTU). At the genus level, the 22 discriminative OTUs of PSD mainly belong to *Blautia* (2 OTU), *Streptococcus* (2 OTU), *Lactobacillus* (1 OTU), *Bacteroides* (1 OTU), *Veillonella* (1 OTU), *Klebsiella* (1 OTU), *Ralstonia* (1 OTU), *Marvinbryantia* (1 OTU), *Mycoplasma* (1 OTU) and *Enterococcus* (1 OTU) (**Supplementary Table S2**).

### Correlations of Depressive-Like Behaviors With Altered Gut Microbes in PSD

**Figure 5:**
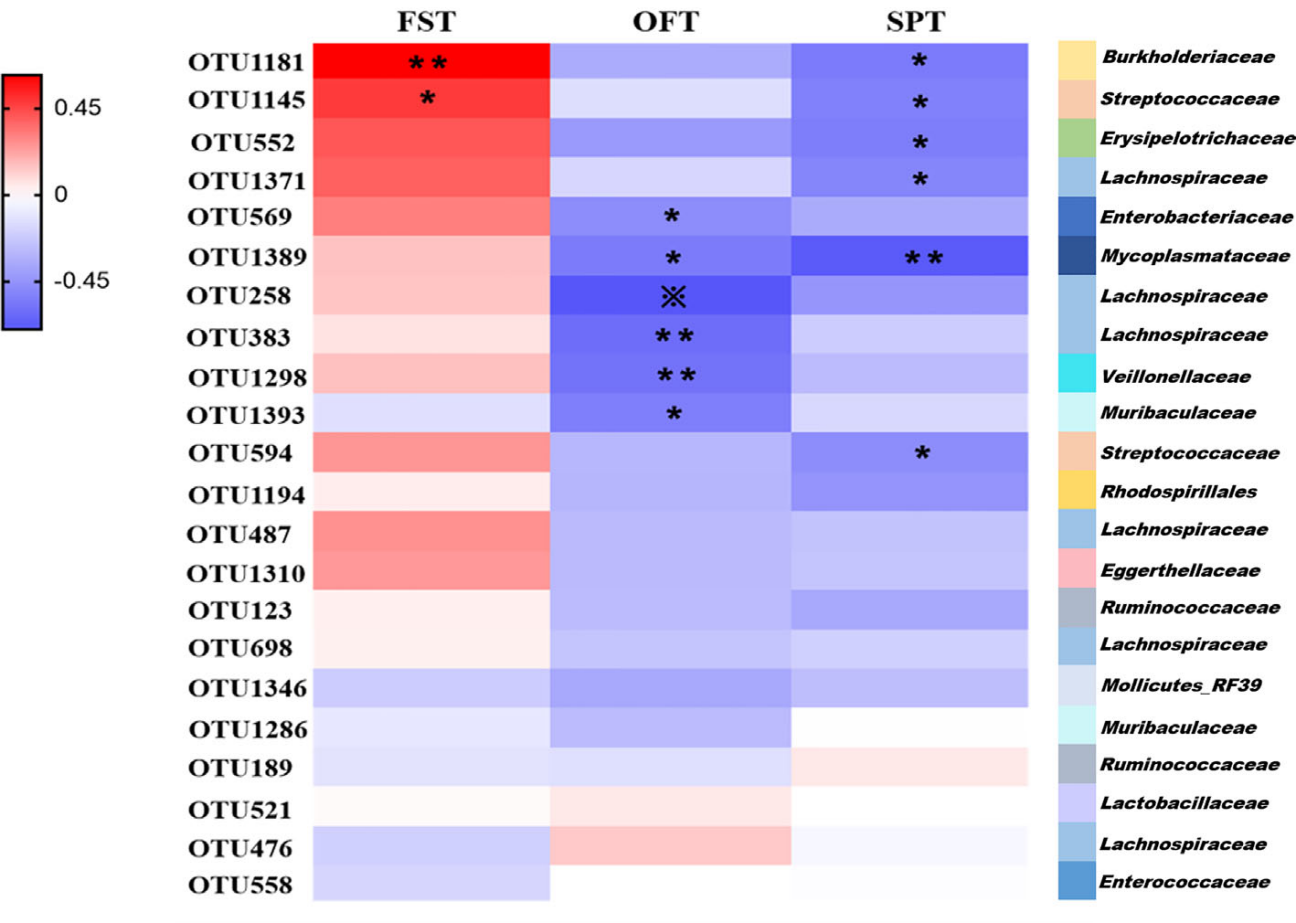
Associations of altered gut microbes with behavior indices.**** Heat map of the Spearman’s rank correlation coefficient of 22 discriminative OTUs for PSD and 3 behavior indices. Red rectangle indicates positive associations between these microbial species and behavior indices, blue rectangle indicates negative associations (n = 6 per group). Overall, 22 discriminative OTUs for PSD were positively associated with FST, and negatively associated with SPT and OFT. 11 of 22 differential microbial variances (50%) were significantly associated with 3 behavior indices (p value < 0.05) and correlation coefficient were ≥0.45 or ≤ −0.45, tested by spearman correlation. The statistical significance was denoted on the rectangle (*p < 0.05; **p < 0.01; ※p < 0.001).

We found that the differential bacterial OTUs were generally associated with behavior indices (**Figure 5**). Overall, the 22 discriminative OTUs of PSD were positively correlated with FST results and negatively correlated with SPT and OFT results. This is consistent with the behavioral test performance of PSD rats. 50% (11/22 OTUs) of altered bacterial OTUs showed significant correlations with FST, OFT and SPT results (r > ± 0.45, p-value < 0.05). Those 11 OTUs were mainly belonging to *Firmicutes* (OTU1145, OTU552, OTU1371, OTU258, OTU383, OTU1298, OTU594), *Proteobacteria* (OTU1181, OTU569), *Bacteroidetes* (OTU1393) and *Tenericutes* (OTU1389). These results indicate that PSD was characterized by disturbed gut microbiome.

### Disturbances of Fecal Metabolisms in PSD Rats

**Figure 6:**
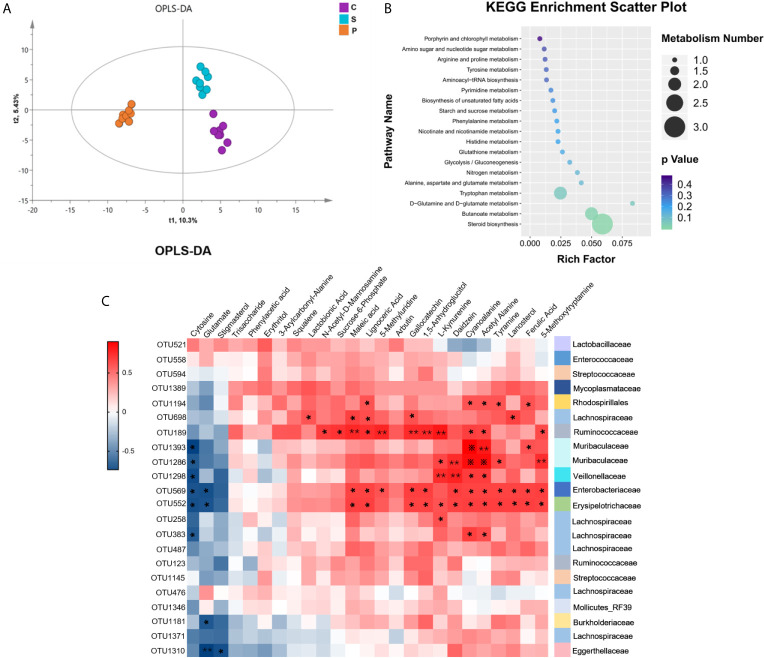
Fecal metabolism characteristics of PSD and its related KEGG enrichment pathways. **(A)** Orthogonal Partial Least Squares Discrimination Analysis (OPLS-DA) showed fecal metabolism of PSD was significantly different from that in control and stroke (n = 8 per group). **(B)** The 25 differential metabolites for PSD rats compared with control and stroke rats were enriched in 19 KEGG pathways. **(C)** Associations of gut microbial OTUs with fecal metabolites. Heat map of the spearman’s rank correlation coefficient of 22 discriminative OTUs and 25 differential metabolites for PSD. Red squares indicate positive associations between these microbial OTUs and metabolites, blue squares indicate negative associations (n = 8 per group). 54.55% (12/22 OTUs) of altered bacterial OTUs showing significant correlations with a range of differential metabolites (p < 0.05) and correlation coefficient were ≥ 0.6 or ≤ −0.6, tested by spearman correlation. The statistical significance was denoted on the squares (*p < 0.05; **p < 0.01; ※p < 0.001).

Considering that the gut microbiota is always involved in the regulation of host’s metabolic pathways, the fecal metabolome is regarded as functional readout of gut microbiome. GC-MS based metabolomic method was used. The fecal metabolic phenotype of PSD was significantly different from that of control and stroke (**Figure 6A**; n = 8 per group). Compared with control and stroke, there were 25 differential fecal metabolites in PSD rats, of which 18 metabolites increased and 7 metabolites decreased (**Supplementary Table S3**, VIP > 1.0 and p-values < 0.05). These differential metabolites were further used for pathway analysis by MetaboAnalyst 4.0. Among 19 pathways revealed, the lipid-related metabolic pathway (steroid biosynthesis) was most significantly enriched (**Figure 6B**, p-values<0.05) (**Supplementary Table S4**). Specifically, these differentially expressed metabolites were related to lipid metabolism (Squalene, Lanosterol, Stigmasterol and Lignoceric Acid), amino acid metabolism (L-Kynurenine, 5-Methoxytryptamine, Tyramine, Glutamate, Maleic acid, Cyanoalanine and Phenylacetic acid), carbohydrate metabolism (Arbutin, N-Acetyl-D-Mannosamine, Glutamate, Acetyl Alanine and Sucrose-6-Phosphate) and nucleotide metabolism (Cytosine).

### Correlations Between Gut Microbes and Fecal Metabolites

To further explore the potential correlations of the altered gut microbial OTUs with fecal metabolome, correlation analysis was performed (**Figure 6C**). 54.55% (12/22 OTUs) of altered bacterial OTUs had a significant correlation with a series of differential metabolites (r > ± 0.6, p-value < 0.05). These 12 OTUs mainly belonged to *Firmicutes* (OTU383, OTU258, OTU552, OTU1298, OTU189, OTU698), *Proteobacteria* (OTU1181, OTU569, OTU1194), *Bacteroidetes* (OTU1286, OTU1393) and *Actinobacteria* (OTU1310). The significantly correlated metabolites were assigned to lipid metabolism (Lanosterol, Stigmasterol and Lignoceric Acid), amino acid metabolism (L-Kynurenine, 5-Methoxytryptamine, Tyramine, Glutamate, Cyanoalanine and Maleic acid), carbohydrate metabolism (N-Acetyl-D-Mannosamine, Glutamate, Acetyl Alanine and Sucrose-6-Phosphate), nucleotide metabolism (Cytosine), biosynthesis of other secondary metabolites (Daidzein, Ferulic Acid and Gallocatechin) and metabolites without metabolic pathways (Lactobionic Acid, 5-Methyluridine and 1,5-Anhydroglucitol). Our findings demonstrated that PSD rats were characterized by both disturbed gut microbiome and fecal metabolome, and altered gut microbiota can affect the metabolism of PSD rats.

## Discussion

In this study, we compared the gut microbiome and fecal metabonomics among the PSD, control and stroke rats. We found that the microbial phenotype of PSD rats was significantly different from that of the control and stroke groups. Importantly, the altered gut microbial OTUs were highly correlated with a series of metabolites. Moreover, the altered gut microbes of PSD rats were highly consistent with their behavioral performance. Enrichment analysis further uncovered the crucial metabolic pathways related to PSD. Our findings suggest that gut microbiome may participate in the development of PSD, and the mechanism may be related to the regulation of lipid metabolism.

One of the problems faced by post-stroke depression research, is the lack of model that highly simulates the clinical disease. The PSD model in our study, is one of the most commonly used and widely accepted models in the world. Previous studies have found that it can partially reflect the pathophysiological mechanism of post-stroke depression (Pang et al., 2015; Zhang et al., 2017; Villa et al., 2018). It should be noted that all rats in our study were housed in a specific pathogen free (SPF) environment with unidirectional flow. The water was purified. The food and padding were sterilized. Therefore, the soiled cage was mainly served as a stress factor in our study, with no substantial impact on the experimental results.

Compared with the control, the microbial composition of PSD showed decreased species richness indices (**Figure 3A**). The diversity of the human gut microbiota is considered evidence of health (Eckburg et al., 2005). Herein, decreased species richness in PSD rats may suggest disordered physiological processes. The gut microbiome of PSD rats has not been explored before, however, previous study on depression showed disturbances of *Lachnospiraceae*, *Lactobacillaceae*, *Streptococcaceae*, *Erysipelotrichaceae* and *Ruminococcaceae* (Zheng et al., 2016). We found similar microbes, such as *Lachnospiraceae*, *Lactobacillaceae*, *Streptococcaceae*, *Erysipelotrichaceae*, *Ruminococcaceae*, *Veillonellaceae*, *Enterococcaceae*, *Muribaculaceae*, *Burkholderiaceae*, *Enterobacteriaceae*, *Mycoplasmataceae* and *Eggerthellaceae* were altered in PSD rats. Unlike PSD and depression, Lee et al. reported that the disturbance of gut microbiome during stroke related to *Bifidobacteriaceae* and *Clostridiaceae* (Lee et al., 2020). Furthermore, the altered bacterial OTUs were highly consistent with the behavioral test performance of PSD rats (**Figure 5**), suggesting the abnormal microbial state of PSD has been verified on the behavioral level. Specifically, our findings showed OTU569 which belongs to *Enterobacteriaceae*, had a significant correlation with both OFT and Lignoceric Acid (**Figures 5** and **6C**). Lignoceric Acid, one of the metabolites detected in our study, was involved in lipid metabolism and also significantly related to *Enterobacteriaceae*. Studies showed that lignoceric acid was associated with increased risk of cardioembolic stroke (Chung et al., 2015) and autoimmune diseases (Tsoukalas et al., 2019). As is known, neuroimmune was involved in central nervous system (CNS) disorders, including PSD (Cacabelos et al., 2016).

We also found that PSD was associated with disturbances of fecal metabolomics. These discriminating fecal metabolites in PSD rats were mainly involved in lipid metabolism, amino acid metabolism, carbohydrate metabolism and nucleotide metabolism. These overlap with the metabolic pathways of depression and stroke. A previous study showed that depressed mice were characterized by disturbances in carbohydrate and amino acid metabolism (Zheng et al., 2016). Another study in depressed cynomolgus macaque demonstrated disruption of carbohydrate and lipid metabolism (Qin et al., 2019). Stroke was also found to be related to disturbances in amino acid and lipid metabolism (Yamashiro et al., 2017; Chen et al., 2019).

Interestingly, a further analysis revealed two lipid-related metabolic pathways in PSD, mainly involved in biosynthesis of steroid and unsaturated fatty acids. Among them, the steroid biosynthetic pathway is significantly enriched, with the highest enrichment density. As is known, cholesterol is the source of steroid-related hormone biosynthesis and is closely related to brain development and neurological diseases (Hussain et al., 2019). Cholesterol can either be converted into steroid-related hormones (estrogens, androgens, glucocorticoids) or vitamin D. On one hand, clinical study found that PSD patients had significantly lower vitamin D levels than non-PSD patients (Han et al., 2015). For depressive patients, low high-density lipoprotein (HDL) cholesterol and high triglyceride levels were associated with lower likelihood of long-term symptom resolution (Virtanen et al., 2017). On the other hand, both clinical and animal studies showed that corticosteroids increased the risk of depression (Weina et al., 2018). Elevated cortisol after stroke was even associated with morbidity and mortality of the patients (Barugh et al., 2014). In our study, of the three metabolites enriched in the steroid pathway, Lanosterol and Squalene were significantly increased, while Stigmasterol was significantly decreased. Lanosterol and Squalene will eventually be converted into cholesterol. Stigmasterol reduces the level of low-density lipoprotein (LDL) cholesterol. Therefore, lipid metabolism and PSD may be closely related. Abnormal lipid metabolism may provide novel clues for investigating the pathogenesis of PSD.

Our research has some limitations. (i) Although we provide evidence that the gut microbiota imbalance may be related to PSD, fecal transplantation experiments can be used to confirm the causality. (ii) Our research was only carried out in male rats. Female rats may also be of interest in future experiments. (iii) Due to the relatively limited resolution of the 16S rRNA sequencing technique (Hillmann et al., 2018), shotgun metagenomic sequencing method will be used to identify specific bacterial strains of PSD. (iv) Based on the identified metabolic pathways related to PSD, it is necessary to further explore their key regulatory targets.

In summary, using multi-omics data, we outlined the landscapes of bacteria as well as fecal metabolites in PSD rats. We found gut microbiome may participate in the development of PSD, the mechanism may be related to the regulation of lipid metabolism. Our findings provide a new perspective for understanding the pathogenesis of PSD.

## Data Availability Statement

The original contributions presented in the study are included in the article/**Supplementary Material**, further inquiries can be directed to the corresponding author.

## Ethics Statement

The animal study was reviewed and approved by the Ethics Committee of Chongqing Medical University (Permit No. 2020–459).

## Author Contributions

Conception and design: JM. Performed the experiments: WJ, LG, FL and YR. Performed the microbiome and metabolomic analysis: WJ. Drafted the manuscript: WJ. Final Approval of the Completed Manuscript: JM. All authors contributed to the article and approved the submitted version.

## Funding

This article was funded by National Natural Science Foundation of China (Grant No. 81371310), Science and Technology Committee of Chongqing (Grant No. cstc2018jcyjAX0130) and Chongqing Health Commission (Grant No. 2020MSXM038).

## Conflict of Interest

The authors declare that the research was conducted in the absence of any commercial or financial relationships that could be construed as a potential conflict of interest.
