# Supplementary material for: Alteration of Gut Microbiome and Correlated Lipid Metabolism in Post-Stroke Depression
Source: Front Cell Infect Microbiol. 2021 Apr 22;11:663967. doi: 10.3389/fcimb.2021.663967 (PMC8100602; doi:10.3389/fcimb.2021.663967)
Supplement: Supplementary file 1 [file DataSheet_1.pdf]

Supplementary Material

Supplementary Tables

Table S1. Stressors used in the CUMS procedure

Week	Monday	Tuesday	Wednesday	Thursday	Friday	Saturday	Sunday
**Week 1**	Water and food deprivation (24hrs)	45° cage tilt (24hrs)	Tail pinch (1min);<br><br>Overnight lighting (36hrs)	Swimming in 4 °C water (5min);<br><br>Soiled cage (24hrs)	Restrain (4hrs);<br><br>Water deprivation (24hrs)	SPT (1hr);<br><br>Food deprivation (24hrs)	Collect fecal samples；<br><br>BW
**Week 2**	Tail pinch (1min)	45° cage tilt (24hrs)	Restrain (4hrs);<br><br>Overnight lighting (36hrs)	Swimming in 4 °C water (5min);<br><br>Soiled cage (24hrs)	Water deprivation (24hrs)	SPT (1hr);<br><br>Food deprivation (24hrs)	Collect fecal samples;<br><br>BW
**Week 3**	Swimming in 4 °C water (5min);<br><br>Soiled cage (24hrs)	Tail pinch (1min)	Restrain (4hrs);<br><br>Overnight lighting (36hrs)	45° cage tilt (24hrs)	Water deprivation (24hrs)	SPT (1hr);<br><br>Food deprivation (24hrs)	Collect fecal samples;<br><br>BW
**Week 4**	Tail pinch (1min)	Soiled cage (24hrs)	Swimming in 4 °C water (5min);<br><br>45° cage tilt (24hrs)	Restrain (4hrs);<br><br>Overnight lighting (36hrs)	Water deprivation (24hrs)	SPT (1hr);<br><br>Food deprivation (24hrs)	Collect fecal samples;<br><br>BW;

SPT, sucrose preference test; BW, body weight

Table S2. Differential operational taxonomic units (OTUs) of PSD

OTU ID	Taxonomic Assignment			Relative Abundance						LDA value	LDA p-value
	Phylum	Family	Genus	C (Mean)	C (Sd)	S (Mean)	S (Sd)	P (Mean)	P (Sd)		
OTU258	Firmicutes	Lachnospiraceae	Blautia	1.4E-03	1.5E-03	5.1E-03	1.2E-02	4.8E-02	6.0E-02	2.40	0.040
OTU383	Firmicutes	Lachnospiraceae	Blautia	5.6E-03	9.8E-03	4.3E-02	6.3E-02	1.1E-01	1.3E-01	2.75	0.044
OTU476	Firmicutes	Lachnospiraceae	Bacteroides	3.3E-02	2.8E-02	0.0E+00	0.0E+00	8.0E-02	1.0E-01	2.63	0.026
OTU487	Firmicutes	Lachnospiraceae	Lachnospiraceae_UCG-001	1.9E-03	3.4E-03	0.0E+00	0.0E+00	2.5E-02	2.3E-02	2.39	0.006
OTU698	Firmicutes	Lachnospiraceae	unclassified_f__Lachnospiraceae	9.3E-04	1.4E-03	0.0E+00	0.0E+00	1.3E-02	1.5E-02	2.30	0.006
OTU1371	Firmicutes	Lachnospiraceae	Marvinbryantia	4.6E-03	3.4E-03	3.6E-02	6.0E-02	6.8E-02	1.0E-01	2.64	0.036
OTU521	Firmicutes	Lactobacillaceae	Lactobacillus	8.8E-03	1.1E-02	0.0E+00	0.0E+00	9.7E-03	1.2E-02	2.16	0.025
OTU594	Firmicutes	Streptococcaceae	Streptococcus	2.5E-02	2.5E-02	1.8E-02	1.4E-02	9.5E-02	6.4E-02	2.66	0.023
OTU1145	Firmicutes	Streptococcaceae	Streptococcus	9.3E-03	1.0E-02	8.8E-03	1.2E-02	3.8E-02	3.9E-02	2.34	0.050
OTU552	Firmicutes	Erysipelotrichaceae	norank_f__Erysipelotrichaceae	0.0E+00	0.0E+00	4.2E-03	5.8E-03	1.4E-02	9.8E-03	2.36	0.013
OTU123	Firmicutes	Ruminococcaceae	Ruminococcaceae_UCG-014	2.8E-03	3.0E-03	1.4E-03	2.3E-03	1.1E-02	8.4E-03	2.52	0.034
OTU189	Firmicutes	Ruminococcaceae	Ruminococcaceae_UCG-014	2.3E-03	2.1E-03	9.3E-04	1.4E-03	6.0E-03	5.4E-03	2.78	0.046
OTU1298	Firmicutes	Veillonellaceae	Veillonella	9.3E-04	2.3E-03	4.6E-04	1.1E-03	2.0E-02	2.5E-02	2.59	0.003
OTU558	Firmicutes	Enterococcaceae	Enterococcus	2.9E-02	3.0E-02	1.4E-03	1.5E-03	5.5E-02	7.8E-02	2.38	0.021
OTU1393	Bacteroidetes	Muribaculaceae	norank_f__Muribaculaceae	4.6E-04	1.1E-03	1.2E-02	2.2E-02	1.0E-01	1.5E-01	2.83	0.029
OTU1286	Bacteroidetes	Muribaculaceae	norank_f__Muribaculaceae	4.6E-04	1.1E-03	3.7E-03	9.1E-03	3.9E-02	5.6E-02	2.46	0.021
OTU1181	Proteobacteria	Burkholderiaceae	Ralstonia	9.3E-04	2.3E-03	4.2E-03	3.8E-03	1.1E-02	8.9E-03	2.32	0.021
OTU569	Proteobacteria	Enterobacteriaceae	Klebsiella	0.0E+00	0.0E+00	9.3E-04	1.4E-03	2.3E-02	2.1E-02	2.50	0.006
OTU1194	Proteobacteria	norank_o__Rhodospirillales	norank_o__Rhodospirillales	0.0E+00	0.0E+00	9.3E-04	1.4E-03	1.3E-02	2.0E-02	2.34	0.040
OTU1346	Tenericutes	norank_o__Mollicutes_RF39	norank_o__Mollicutes_RF39	1.7E-02	2.2E-02	7.4E-03	1.8E-02	4.5E-02	3.8E-02	2.37	0.027
OTU1389	Tenericutes	Mycoplasmataceae	Mycoplasma	4.6E-04	1.1E-03	4.6E-04	1.1E-03	5.6E-03	8.2E-03	2.42	0.008
OTU1310	Actinobacteria	Eggerthellaceae	unclassified_f__Eggerthellaceae	1.2E-02	8.3E-03	1.9E-02	1.4E-02	3.5E-02	1.7E-02	2.25	0.046

C, Control; S, Stroke; P, post-stroke depression; Sd, standard deviation

Table S3. Discriminative fecal metabolites of PSD

Metabolite name	Relative Abundance								up/down regulation
	C (mean)	C (SD)	S (mean)	S (SD)	PSD (mean)	PSD (SD)	FC(PSD/C)	FC(PSD/S)	
Arbutin	1.74E-05	8.30E-06	1.56E-05	6.40E-06	6.39E-04	4.64E-04	36.67	40.91	↑
Lactobionic Acid	3.02E-05	1.45E-05	1.01E-04	1.93E-04	9.68E-04	1.05E-03	32.02	9.59	↑
Gallocatechin	4.94E-05	3.59E-05	5.86E-05	2.97E-05	1.43E-03	1.36E-03	28.99	24.45	↑
N-Acetyl-D-Mannosamine	2.89E-05	1.36E-05	2.52E-05	8.49E-06	7.17E-04	6.25E-04	24.79	28.45	↑
5-Methyluridine	4.62E-05	4.11E-05	2.65E-05	1.19E-05	1.03E-03	1.01E-03	22.19	38.69	↑
1,5-Anhydroglucitol	2.75E-05	1.31E-05	2.34E-05	6.55E-06	6.02E-04	5.30E-04	21.91	25.76	↑
Lanosterol	4.88E-05	1.85E-05	2.05E-04	4.44E-04	1.02E-03	8.98E-04	20.86	4.98	↑
Sucrose-6-Phosphate	4.95E-05	3.92E-05	5.72E-05	3.57E-05	6.84E-04	8.79E-04	13.81	11.97	↑
Maleic acid	5.68E-05	3.06E-05	3.76E-05	1.62E-05	7.53E-04	8.02E-04	13.27	20.00	↑
Tyramine	1.13E-04	4.16E-05	1.01E-04	7.37E-05	9.29E-04	7.58E-04	8.22	9.19	↑
Cyanoalanine	2.28E-04	3.84E-04	6.89E-04	6.54E-04	1.59E-03	7.48E-04	6.97	2.31	↑
Ferulic Acid	1.37E-04	1.10E-04	1.85E-04	2.58E-04	8.98E-04	8.73E-04	6.57	4.86	↑
L-Kynurenine	1.52E-04	3.67E-04	1.53E-04	3.76E-04	9.52E-04	5.07E-04	6.25	6.20	↑
Daidzein	1.29E-04	2.22E-04	1.62E-04	2.78E-04	5.86E-04	3.32E-04	4.55	3.62	↑
Acetyl Alanine	3.03E-04	5.21E-04	3.15E-04	5.44E-04	1.22E-03	8.80E-04	4.03	3.88	↑
5-Methoxytryptamine	1.28E-04	2.64E-04	5.59E-05	3.47E-05	5.03E-04	3.23E-04	3.94	9.01	↑
Lignoceric Acid	3.68E-04	2.36E-04	5.17E-04	2.70E-04	8.32E-04	2.30E-04	2.26	1.61	↑
Squalene	2.65E-03	1.01E-03	2.59E-03	9.62E-04	2.79E-03	8.90E-04	1.06	1.08	↑
Trisaccharide	8.32E-05	4.80E-05	8.49E-05	3.25E-05	6.23E-05	3.46E-05	0.75	0.73	↓
Phenylacetic acid	2.04E-03	1.25E-03	1.70E-03	9.05E-04	1.12E-03	5.93E-04	0.55	0.66	↓
3-Arylcarbonyl-Alanine	5.69E-04	4.48E-04	5.95E-04	2.90E-04	3.11E-04	2.29E-04	0.55	0.52	↓
Erythritol	1.82E-04	8.12E-05	1.30E-04	5.16E-05	9.62E-05	5.23E-05	0.53	0.74	↓
Glutamate	9.28E-04	2.56E-04	8.55E-04	4.51E-04	4.78E-04	1.45E-04	0.52	0.56	↓
Cytosine	6.97E-04	3.56E-04	6.59E-04	2.90E-04	3.36E-04	1.30E-04	0.48	0.51	↓
Stigmasterol	1.89E-03	1.09E-03	1.62E-03	3.86E-04	6.66E-04	3.71E-04	0.35	0.41	↓

C, Control; S, Stroke; FC, Fold Change; SD, standard deviation; ↑up regulation; ↓down regulation

Table S4. KEGG pathway analysis

KEGG_ID	Pathway Name	KEGG Compound	Enriched Metabolites	p-value
ko00100	Steroid biosynthesis	C00751, C01724, C05442	3	0.0017
ko00650	Butanoate metabolism	C00025, C01384	2	0.0156
ko00471	D-Glutamine and D-glutamate metabolism	C00025	1	0.0572
ko00380	Tryptophan metabolism	C00328, C05659	2	0.0578
ko00250	Alanine, aspartate and glutamate metabolism	C00025	1	0.1110
ko00010	Glycolysis / Gluconeogenesis	C06186	1	0.1420
ko00910	Nitrogen metabolism	C00025	1	0.1200
ko00480	Glutathione metabolism	C00025	1	0.1710
ko00340	Histidine metabolism	C00025	1	0.1950
ko00760	Nicotinate and nicotinamide metabolism	C01384	1	0.1950
ko00360	Phenylalanine metabolism	C07086	1	0.2030
ko00500	Starch and sucrose metabolism	C16688	1	0.2190
ko01040	Biosynthesis of unsaturated fatty acids	C08320	1	0.2340
ko00240	Pyrimidine metabolism	C00380	1	0.2530
ko00970	Aminoacyl-tRNA biosynthesis	C00025	1	0.3110
ko00350	Tyrosine metabolism	C00483	1	0.3140
ko00330	Arginine and proline metabolism	C00025	1	0.3340
ko00520	Amino sugar and nucleotide sugar metabolism	C00645	1	0.3510
ko00860	Porphyrin and chlorophyll metabolism	C00025	1	0.4680
